# Artesunate Reduces Serum Lipopolysaccharide in Cecal Ligation/Puncture Mice via Enhanced LPS Internalization by Macrophages through Increased mRNA Expression of Scavenger Receptors

**DOI:** 10.3390/ijms15011143

**Published:** 2014-01-16

**Authors:** Bin Li, Mengchen Yu, Xichun Pan, Chuanliang Ren, Wei Peng, Xiaoli Li, Weiwei Jiang, Jiang Zheng, Hong Zhou

**Affiliations:** 1 Department of Pharmacology, College of Pharmacy, Third Military Medical University, Chongqing 400038, China; E-Mails: libin6033@sina.com (B.L.); agnes15@163.com (M.Y.); xichunpan@163.com (X.P.); renchuanliang123@126.com (C.R.); pengwei002@126.com (W.P.); plum181181@126.com (X.L.); jww613@sina.com (W.J.); 2 Medical Research Center, Southwestern Hospital, Third Military Medical University, Chongqing 400038, China

**Keywords:** CLP, artesunate, macrophages, LPS internalization, scavenger receptor

## Abstract

Innate immunity is the first line of defense in human beings against pathogen infection; monocytes/macrophages are the primary cells of the innate immune system. Recently, macrophages/monocytes have been discovered to participate in LPS clearance, and the clearance efficiency determines the magnitude of the inflammatory response and subsequent organ injury. Previously, we reported that artesunate (AS) protected sepsis mice against heat-killed *E. coli* challenge. Herein, we further confirmed that AS protected cecal ligation/puncture (CLP) sepsis mice. Its protection on sepsis mice was related to not only reduction of pro-inflammatory cytokines and serum LPS levels but also improvement of liver function. Based on the fact that AS did not directly bind and neutralize LPS, we hypothesized that the reduction of serum LPS level might be related to enhancement of LPS internalization and subsequent detoxification. Our results showed that AS increased FITC-LPS internalization by peritoneal macrophage and liver Kupffer cell, but enhancement of LPS internalization by AS was not related to the clathrin-dependent pathway. However, AS induced mRNA expression of important scavenger receptors (SRs); *SR-A* and *MARCO* mRNA expression was upregulated, suggesting that AS enhancement of LPS internalization and inhibition of pro-inflammatory cytokines was related to changes in mRNA expression of SRs.

## 1. Introduction

Sepsis is a progressive and life-threatening clinical syndrome that is characterized by an exaggerated inflammatory and innate immune response to infections. Despite improvements in supportive care, there is no improvement of hospital mortality over the past decade [1].

The defensive response to sepsis involves the engagement of the innate immune system. This system attempts to remove invading microorganisms and elicits a robust acute inflammatory response. Within the innate immune system, monocytes/macrophages play a very important role during the immune response through the recognition of pathogens and the release of pro-inflammatory cytokines. For monocytes/macrophages, internalization is a fundamental cellular process and is an essential step in host defense against bacteria.

Lipopolysaccharide/endotoxin (LPS) is a well-recognized pathogenic factor that induces sepsis and is thus regarded as an important drug target. LPS can be recognized by monocytes/macrophages and then induce the release of large amounts of pro-inflammatory cytokines. Previously, LPS internalization was found to be the first and primary process of liver cell detoxification [2,3]. Interestingly, recently LPS clearance has been discovered within macrophages/monocytes [4], suggesting macrophages/monocytes may be involved in LPS detoxification.

Artesunate (AS) is a water-soluble hemisuccinate derivative of dihydroartemisinin. In our previous study, AS was found to protect sepsis model mice challenged with heat-killed *Escherichia coli* (*E. coli*). The protection was closely related to the reduction of serum LPS and pro-inflammatory cytokines levels in sepsis model mice [5]. However, AS itself could not directly bind and neutralize LPS, suggesting the protection through AS on sepsis model mice was not related to its direct neutralization of LPS [5]. Therefore, we wondered whether the reduction of serum LPS produced by AS was related to enhancement of LPS internalization and subsequent detoxification.

Therefore, in the present experiments the effect of AS on cecal ligation/puncture (CLP) mice model was firstly observed, and then the influence of AS on LPS internalization from peritoneal macrophage and liver Kupffer cells was investigated *in vitro*. Finally, the possible molecular mechanism was further investigated.

## 2. Results and Discussion

### 2.1. AS Protection on CLP Mice Is Related to Decreasing Serum Cytokines and LPS Levels

#### 2.1.1. AS Improves the Survival of CLP Mice

**Figure 1.**
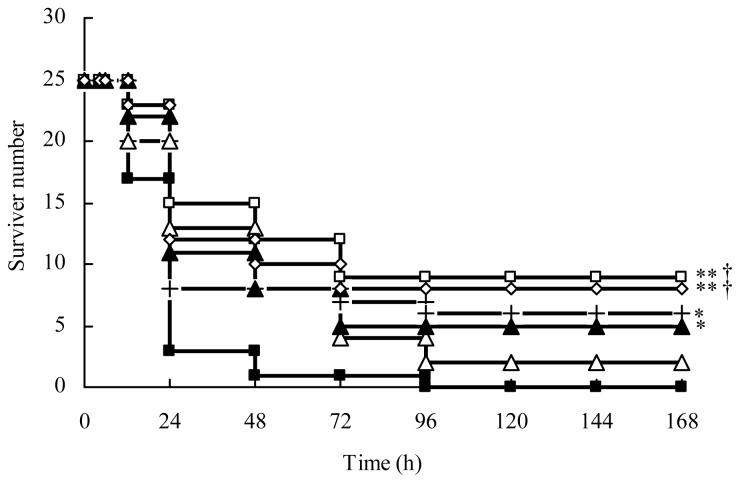
Protection of AS in combination with AMPS on CLP mice. One hundred and fifty mice was randomly divided into six groups (*n* = 25) and treated as follows: ■, CLP group; +, CLP and AS (30 mg/kg) alone; △, CLP and AMPS (100 mg/kg); ⋄, CLP and AS (7.5 mg/kg) in combination with AMPS; □, CLP and AS (15 mg/kg) in combination with AMPS; and ▲, CLP and AS (30 mg/kg) in combination with AMPS. AS and AMPS was repeatedly administered at 0, 4, 24, and 48 h after CLP operation. The mice were fed *ad libitum* and the mortality was observed for seven days. * *p* < 0.05, ** *p* < 0.01 as compared to CLP group; † *p* < 0.05 as compared to CLP and AMPS group. Other details are as described under Materials and Methods.

Almost all CLP sepsis model mice died within four days. The mice treated with 100 mg/kg of ampicillin sodium-sulbactam sodium (AMPS) received only eight percent protection within seven days. The mice immediately treated with only AS (30 mg/kg) after CLP received 24.0% protection and the time of death was obviously delayed. Significantly, AS (7.5, 15 and 30 mg/kg) in combination with AMPS (100 mg/kg) provided protection of 32.0%, 36.0%, and 20.0%, respectively (Figure 1). These results demonstrated both AS alone and AS in combination with AMPS provided significant protection against CLP sepsis mice.

#### 2.1.2. AS Decreases the Serum Pro-Inflammatory Cytokine Levels of CLP Mice

**Figure 2.**
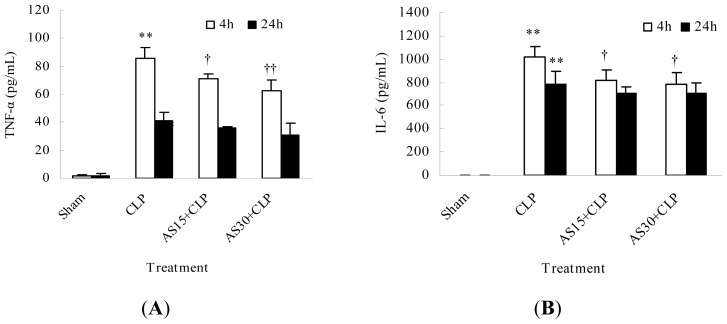
Effects of AS on serum TNF-α (**A**) and IL-6 (**B**) of CLP mice. Thirty-two mice were randomly divided into four groups (*n* = 8). AS was repeatedly administered at 0 and 4 h after CLP operation. At 4 and 24 h after operation, four mice from each group were sacrificed, respectively. Sera were separated from the mice by centrifugation at 3500× *g* for 20 min and stored at −80 °C for subsequent cytokine assays. TNF-α or IL-6 level was assayed using respective mouse ELISA kit. ** *p* < 0.01 as compared to Sham group; † *p* < 0.05, †† *p* < 0.01 as compared to CLP group. Other details are as described under Materials and Methods.

Pro-inflammatory cytokines act as good indicators of sepsis. Among pro-inflammatory cytokines, TNF-α and IL-6 are regarded as early and late cytokines, respectively [6]. Herein, AS alone was administrated in order to observe its influence on cytokines release of CLP mice. The results showed serum TNF-α and IL-6 levels sharply increased in CLP model mice compared with those in sham group. AS (15 and 30 mg/kg) treatment obviously decreased serum TNF-α and IL-6 levels at 4 h but not at 24 h after CLP operation (Figure 2). These results suggested AS protection on CLP mice was related to the reduction of pro-inflammatory cytokines.

#### 2.1.3. AS Reduces the Serum LPS Level of CLP Mice

**Figure 3.**
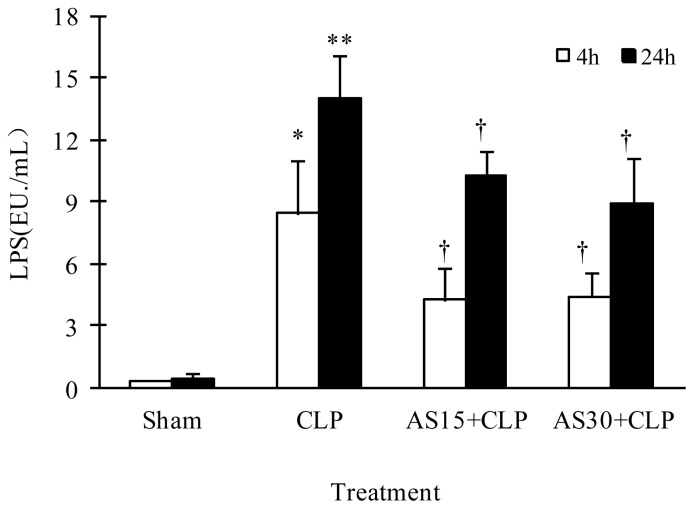
Effect of AS on serum LPS level of CLP models. Thirty-two mice were randomly divided into four groups (*n* = 8). AS was repeatedly administered at 0 and 4 h after CLP operation. At 4 and 24 h after operation, four mice from each group were sacrificed, respectively. Sera were separated from the mice by centrifugation at 3500× *g* for 20 min and stored at −80 °C for subsequent assay. The serum LPS level was assayed using limulus amebocyte lysate (LAL) test. * *p* < 0.05, ** *p* < 0.01 as compared to Sham group; † *p* < 0.05 as compared to CLP group. Other details are as described under Materials and Methods.

LPS level is closely related to pro-inflammatory cytokine level and the outcome of sepsis patients [7]. Herein, our results showed serum LPS level of CLP model mice markedly increased compared with that in the sham group. AS (15 and 30 mg/kg) treatment significantly decreased LPS level in CLP mice at both 4 and 24 h after CLP (Figure 3). The result suggested AS protection on CLP mice was related to the reduction of LPS level.

#### 2.1.4. AS Protects Liver Function of CLP Mice

**Figure 4.**
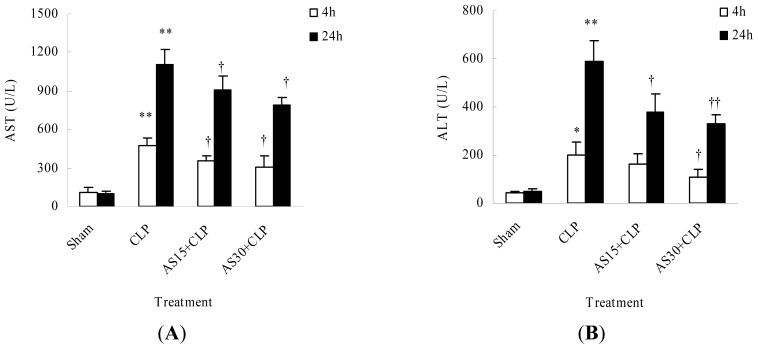
Effect of AS on serum AST (**A**) and ALT (**B**) activity of CLP mice. Thirty-two mice were randomly divided into 4 groups. At 4 and 24 h after operation, four mice from each group were sacrificed, respectively. Sera were separated from the blood samples by centrifugation, aliquoted and stored at −80 °C for subsequent enzyme activity assay. Serum activity of AST and ALT were measured using spectrophotometric diagnostic kits. * *p* < 0.05, ** *p* < 0.01 as compared to the Sham group; † *p* < 0.05 and †† *p* < 0.01 as compared to CLP group. Other details are as described under Materials and methods.

The liver is the most important metabolic organ and its function affects animal survival. For LPS, liver is the major metabolic location [8]. Improved LPS metabolism is helpful to animal survival in sepsis. To clarify that AS protection on sepsis mice was related to liver function, AS influence on liver function was investigated. Herein the results showed that liver alanine aminotransferase (ALT) and aspartate aminotransferase (AST), two markers of liver function, sharply increased in CLP model mice compared with those of sham group. AS (15 and 30 mg/kg) treatment could significantly inhibit the increase of AST and ALT at 4 and 24 h after CLP operation (Figure 4). This result indicated that AS protection is related to the protection of liver function.

### 2.2. AS Enhancement on LPS Internalization Is not Related to the Clathrin-Dependent Pathway

#### 2.2.1. AS Increases FITC-LPS Internalization by Peritoneal Macrophages and Liver Kupffer Cells

**Figure 5.**
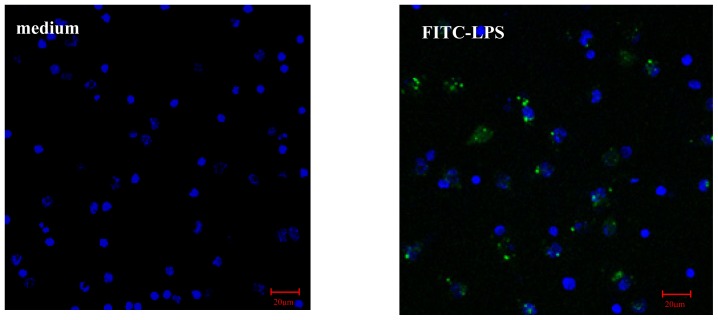

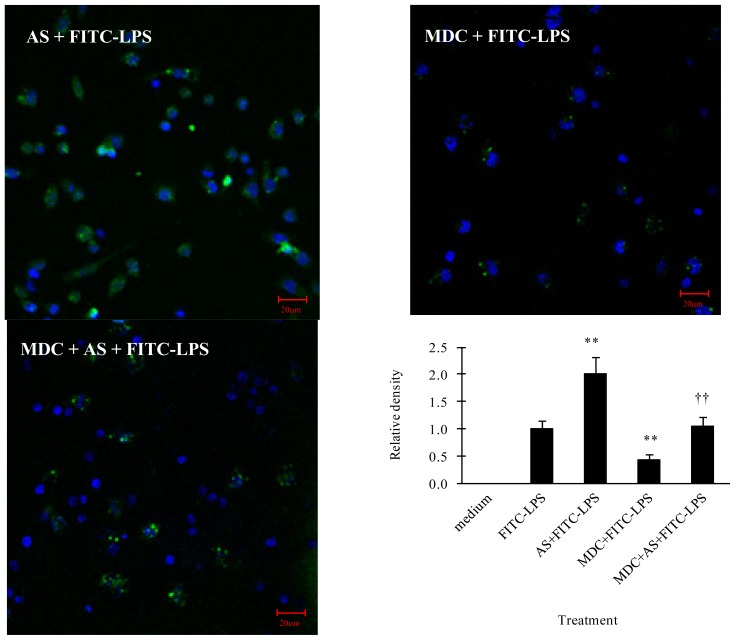
Effect of AS on LPS internalization by mouse peritoneal macrophages (400×). Mouse peritoneal macrophages (1.0 × 10^6^/mL, 500 μL) grown on glass coverslips were pretreated with normal saline (NS) (medium); treated with FITC-LPS (500 ng/mL) for 30 min (FITC-LPS); pretreated with AS (10 μg/mL) for 2 h and then treated with FITC-LPS (500 ng/mL) for 30 min (AS + FITC-LPS); pretreated with MDC (25 μg/mL) and then treated with FITC-LPS (500 ng/mL) for 30 min (MDC + FITC-LPS); pretreated with MDC (25 μg/mL) for 1 h and AS (10 μg/mL) for 2 h, and then treated with FITC-LPS (500 ng/mL) for 30 min (MDC + AS + FITC-LPS). After washing and fixation, the nuclei were stained with 4′,6-diamidino-2-phenylindole (DAPI, blue). The distribution of FITC-LPS as green color in cells was observed by laser confocal microscope. Images were captured and processed using the LSM Image Examiner software. ** *p* < 0.01 as compared to FITC-LPS, †† *p* < 0.01 as compared to MDC + FITC-LPS.

**Figure 6.**
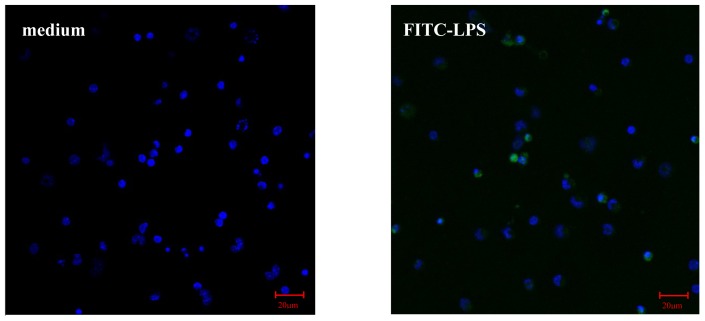

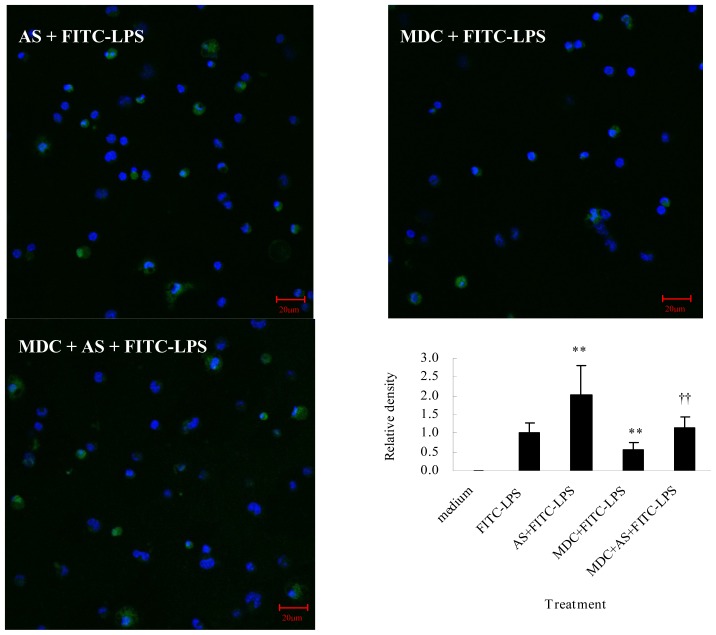
Effect of AS on LPS internalization by mouse liver Kupffer cells (400×). Mouse Kupffer cells (1.0 × 10^6^/mL, 500 μL) grown on glass coverslips were pretreated as described in Figure 5. After washing and fixation, the nuclei were stained with DAPI. The distribution of FITC-LPS in cells was observed by laser confocal microscope. Images were captured and processed using the LSM Image Examiner software. ** *p* < 0.01 as compared to FITC-LPS, †† *p* < 0.01 as compared to MDC + FITC-LPS.

LPS internalization by liver cells is a necessary and important process for the degradation and elimination of LPS from the circulation [3,9]. Recently, LPS clearance has been discovered within macrophages/monocytes, suggesting monocytes/macrophages also participate in LPS clearance [4]. In order to investigate whether AS influence on LPS level was related to the increased LPS internalization of macrophages and subsequent detoxification, the effect of AS on internalization of fluorescein isothiocyanate (FITC-LPS) was observed using confocal microscopy. Herein, the results showed green fluorescence intensity in the mouse peritoneal macrophages or mouse Kupffer cells markedly increased after FITC-LPS treatment for one hour. However, AS further significantly increased green fluorescence intensity of both cells (Figures 5 and 6), suggesting AS could increase the LPS internalization by both peritoneal macrophage and liver Kupffer cells. Furthermore, the results from Kupffer cells from CLP mice showed LPS internalization was obviously enhanced if CLP mice were treated with AS (Figure S1), suggesting AS indeed enhanced LPS internalization.

LPS can be internalized via the clathrin-dependent pathway and then enters the endosome. Therefore a specific inhibitor to disturb clathrin-dependent internalization will inhibit LPS internalization and subsequent pro-inflammatory cytokines release [10–12]. Herein, monodansylcadaverine (MDC), a specific inhibitor for the clathrin-dependent pathway, was used. The results showed MDC pretreatment markedly suppresses LPS internalization by peritoneal macrophages or liver Kupffer cells. However, in the presence of MDC, AS still enhanced LPS internalization by peritoneal macrophages or liver Kupffer cells (Figures 5 and 6), suggesting AS enhancement of LPS internalization is not related to the clathrin-dependent pathway.

#### 2.2.2. AS Inhibits TNF-α and IL-6 Release from Peritoneal Macrophages and Liver Kupffer Cells Induced by LPS

**Figure 7.**
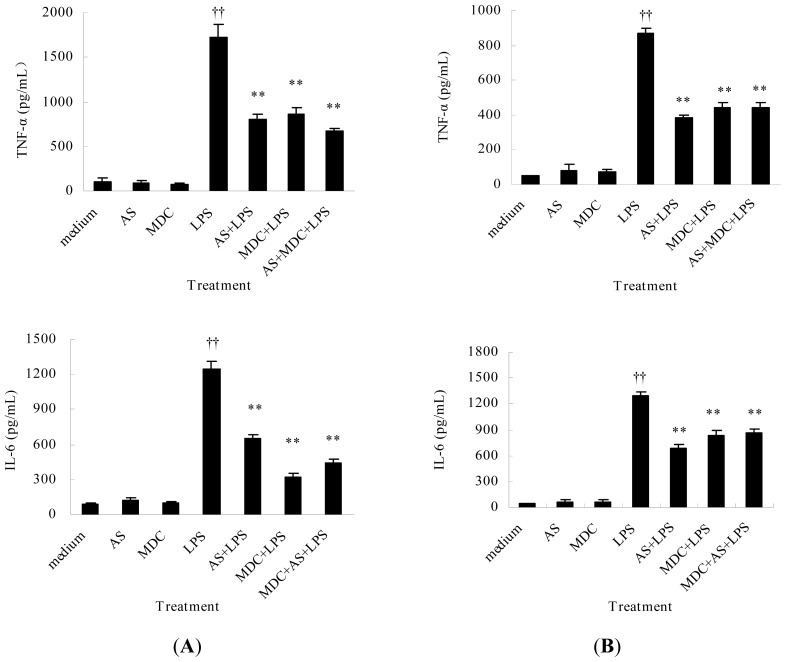
Inhibition of AS on TNF-α and IL-6 release induced by LPS from peritoneal macrophages (**A**) or liver Kupffer cells (**B**). Cells (1.0 × 10^6^ cells/mL, 0.4 mL) were incubated in 48-well plates and co-incubated with MDC (25 μM) for 1 h. Then, cells were pretreated with AS (10 μg/mL) for 2 h and treated with LPS (0.1 μg/mL) for 4 h. The levels of TNF-α and IL-6 in the supernatants were measured using ELISA kits. †† *p* < 0.05 as compared to medium, ** *p* < 0.01 as compared to LPS.

LPS can induce macrophages/monocytes to release large amounts of pro-inflammatory cytokines. However, macrophages/monocytes also play important roles in LPS clearance from the circulation, and the clearance efficiency of LPS determines the magnitude of the inflammatory response and organ injury [4]. In *in vivo* experiments, AS could strongly decrease the serum pro-inflammatory cytokines levels in CLP mice. Herein, the results showed LPS induces large amounts of TNF-α and IL-6 release from both peritoneal macrophages and liver Kupffer cells induced by LPS, and AS significantly inhibited TNF-α and IL-6 release (Figure 7), demonstrating AS decreases pro-inflammatory cytokines levels not only *in vivo* but also *in vitro*. Meanwhile, the results showed MDC pretreatment significantly decreased TNF-α and IL-6 release induced by LPS, too. But MDC could not affect the inhibitory effect of AS on pro-inflammatory cytokine release (Figure 7), further demonstrating that AS enhancement of LPS internalization was not related to the clathrin-dependent pathway.

### 2.3. AS Increases Scavenger Receptors mRNA Expression in Peritoneal Macrophages

**Figure 8.**
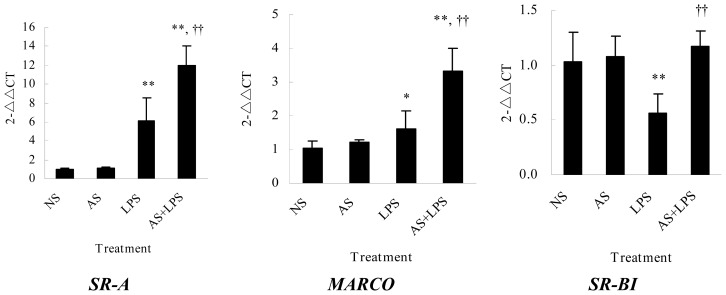
Influences of AS on mRNA expression of *SR-A*, *MARCO* and *SR-BI*. Peritoneal macrophages (2.0 × 10^6^/mL, 2 mL) in six-well plates were incubated with or without AS (10 μg/mL) for 2 h and treated with LPS (100 ng/mL) for 4 h. Total RNA was extracted, and then qPCR was performed. Amplification reactions were performed in a 7500 Real-Time PCR System (Applied Biosystems, Foster City, CA, USA) for 40 cycles of denaturation at 94 °C for 30 s, annealing at 55 °C for 30 s and extension at 72 °C for 40 s. *C*_T_ values were determined using the 7500 System SDS Software (v.1.2.3, Applied Biosystems, Foster City, CA, USA). Expression ratios were finally calculated according to the 2^−△△^*^C^*^T^ method. Data shown were the means ± standard deviation from four independent experiments. * *p* < 0.05, ** *p* < 0.01 as compared with medium, †† *p* < 0.01 as compared with LPS.

Scavenger receptors (SRs) such as *SR-A* (scavenger receptor class A), *MARCO* (macrophage receptor with collagenous structure) and *SR-BI* (scavenger receptor class B type 1) can mediate LPS internalization [13–15]. Based on the results that AS increased LPS internalization by macrophage cells, *SR-A*, *MARCO* and *SR-BI* mRNA expression in peritoneal macrophages was investigated to determine whether the AS effect was related to its inhibition on mRNA expression of SRs. The results from the quantitative PCR (qPCR) assays showed that LPS increased *SR-A* and *MARCO* mRNA expression and that AS alone had no influence, but AS could further increase *SR-A* and *MARCO* mRNA expression induced by LPS. LPS decreased *SR-BI* mRNA expression and AS alone had no influence, but AS could restore *SR-BI* mRNA expression that was initially inhibited by LPS (Figure 8). These results demonstrated that AS enhancement on LPS internalization and inhibition on pro-inflammatory cytokines was related to changes in SRs mRNA expression.

### 2.4. AS Used in Indicated Concentrations Exhibits no Cellular Toxicity *in Vitro*

**Figure 9.**
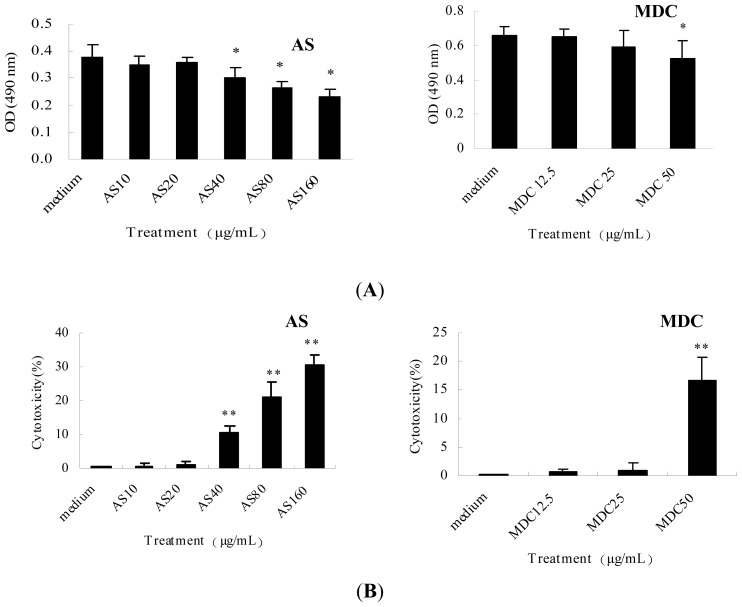
Cytotoxicities of AS and MDC on mouse peritoneal macrophages using MTT (**A**) and LDH (**B**) assay. The peritoneal macrophages (5.0 × 10^4^ cells/well) were grown in 96-well plates. After overnight incubation in RPMI 1640, the cells were washed and incubated with NS or various concentrations of AS or MDC for 6 h. (**A**) Subsequently, 20 μL of MTT solution (5 mg/mL in PBS) was added to the medium in a total volume of 200 μL. After the cells were incubated for 4 or 24 h at 37 °C, the supernatant was removed, and 150 μL DMSO was added to each well to dissolve the produced formazan crystals. The extinction was measured at 490 nm using a microplate reader (Bio-Rad, Hercules, CA, USA); (**B**) After the cells were centrifuged at 400× *g* for 5 min, the clear supernatant (120 μL/well) was transferred into corresponding wells of an optically clear 96-well plate. 60 μL of the LDH reaction mixture was added to each well and incubated for up to 30 min at room temperature (the plate was protected from light). The extinction was measured at 490 nm using a Model 550 microplate reader (Bio-Rad, Hercules, CA, USA). The percentage cytotoxicity was calculated according to the protocol of the manufacturer’s instructions: Cytotoxicity (%) = (Test Sample – Low Control)/(High Control – Low Control) × 100. * *p* < 0.05 *vs.* medium.** *p* < 0.01 *vs.* medium.

In order to exclude the possibility that the effects of AS or MDC on LPS internalization and pro-inflammatory cytokines were due to cytotoxicities, the cellular toxicities of AS and MDC on mice peritoneal macrophages were examined using 3-(4,5)-dimethylthiahiazo(-*Z*-y1)-3,5-diphenytetrazoliumromide (MTT) or lactate dehydrogenase (LDH) cytotoxicity assay. The results showed AS and MDC used in the indicated concentrations had no influence on cell viability; only high concentrations of AS (≥40 μg/mL) and MDC (50 μg/mL) were found to possess slight cellular toxicity for mice peritoneal macrophages (Figure 9). These results demonstrated the effect of AS or MDC is not related to its cellular toxicity.

### 2.5. Discussion

In the present experiments, we found AS protected CLP sepsis mice and this protection was closely related to decreased serum LPS and pro-inflammatory cytokines levels. To the best of our knowledge, this is the first report to demonstrate that reduction of serum LPS produced by AS in CLP mice is related to enhancement of LPS internalization of macrophage via increasing mRNA expression of scavenger receptors

AS is a water-soluble hemisuccinate derivative of dihydroartemisinin. Previously, we reported AS protected sepsis model mice challenged with heat-killed *E. coli* by decreasing pro-inflammatory cytokines levels [5]. In the present experiments, we further demonstrated AS alone or in combination with antibiotics improved CLP mice survival rate. Importantly, the results from the CLP sepsis model further confirmed AS could protect sepsis mice not only in mice challenged by heat-killed *E. coli* but also CLP sepsis mice. In addition to survival rate, AS inhibited the increase of AST and ALT in CLP mice, suggesting that AS protection of sepsis mice was related to improved liver function and LPS metabolism.

The cecal ligation and puncture (CLP) model is a typical self-infection model, which features the onset of sepsis secondary to local abdominal infection originated from the leaked cecum induced by surgery of ligation and puncture of the cecum. Because AS itself has anti-inflammatory activity but no antibacterial ability [9], treatment of AS alone cannot provide enough protection for mice infected with live bacteria, so an antibacterial agent (AMPS in this experiment) is needed to inhibit bacterial growth. According to the clinical dosage regimen of AS against malaria, AS was repeatedly administered at 0, 4, 24, and 48 h after CLP operation. The total dose of AS is not greater than the clinical equivalent dose against malaria. This dosage regimen was introduced into our previous experiments [5,16]. Half-life time of AMPS is very short (1 h); therefore, repeated administration of drug is needed to maintain effective drug concentration. Herein, AMPS was repeatedly administered at 0, 4, 24, and 48 h after CLP operation in keeping with the dosage regimen of AS. This is also not greater than the clinical equivalent dose.

LPS is a strong activator of sepsis via the induction of monocytes/macrophages to release large amounts of pro-inflammatory cytokines [17]. In the present experiments, according to the dosage of AS against malaria, AS was only administrated at 0 and 4 h after CLP operation and AMPS was not administered in order to observe the influence of AS on cytokine release at 4 and 24 h (long-time influence). The results showed AS (15 and 30 mg/kg) reduced serum pro-inflammatory cytokines levels at 4 but not 24 h, which was in line with the short half-life time of AS.

Previously, we found AS decreased serum LPS level of mice challenged by heat-killed *E. coli* [5]. Herein, we found AS decreased serum LPS level in CLP sepsis mice, too. Based on the fact that AS couldn’t directly bind and neutralize LPS [5], we thought AS’s reduction on serum LPS level wasn’t related to its directly binding and neutralizing LPS. Therefore, we supposed the decreased LPS level might be related to enhancement of LPS internalization and subsequent detoxification.

Innate immunity acts as the first line of defense in human beings. Monocytes/macrophages are the primary cells of the innate immune system via recognition of diverse pathogens through pattern recognition receptors (PRR) and the induction of release of pro-inflammatory cytokines. The liver is the primary site of LPS clearance; hepatocytes are the dominant cell for LPS clearance [18]. Recently, LPS clearance has been discovered within macrophages/monocytes, suggesting that monocytes/macrophages also participate in LPS clearance [4]. Herein, our results showed AS could increase LPS internalization by both peritoneal macrophages and liver Kupffer cells, suggesting AS indeed could increase LPS entrance into macrophage and liver Kupffer cells.

LPS can be internalized via cell membrane receptor-mediated internalization. TLR4, a PRR for LPS, is thought to be very important for LPS internalization [10,19]. Absence of TLR4 on the membrane of hepatocytes will lead to decreased LPS clearance [18,20]. The results from our experiments and other experiments confirmed that LPS is internalized via the clathrin-dependent pathway [10,19,21]. MDC, a specific inhibitor for the clathrin-dependent pathway, significantly decreases LPS internalization and subsequent pro-inflammatory cytokines release [21]. Our results showed AS enhancement on LPS internalization was not related to the clathrin-dependent pathway because MDC pretreatment could not affect the inhibitory effect of AS on pro-inflammatory cytokines release. Previous results demonstrated that AS down-regulated TLR4 mRNA expression of peritoneal macrophages treated with LPS [5], therefore, we considered that enhancement of LPS internalization produced by AS was not related to the decreased TLR4 mRNA expression.

It is known that LPS can be internalized via other cell membrane receptor-mediated internalization besides TLR4. It was discovered that TLR4 does not play a key role in cellular LPS internalization by monocytes or endothelial cells; these cell types mainly internalize LPS by a SR pathway during LPS clearance and detoxification [14,22,23]. Inhibited expression of SRs might greatly influence LPS clearance of macrophages *in vivo* [24].

The SR family is a large group of receptors, which have been classified into eight different classes (Class A, B, C, D, E, F, G and H) according to the classification proposed by Krieger and coworkers [25]. Among these receptors, *SR-A* is very important. *SR-A* participates in LPS internalization and then clears extracellular LPS. Importantly, *SR-A* competitively inhibits LPS binding to TLR4, resulting in the suppression of TLR4-mediated signaling [13,26]. *MARCO* is another distinct member of the class A SR families, acting as a phagocytic receptor to bind LPS [14,27]. *SR-BI*, another important scavenger receptor, can bind and uptake LPS [15], exerting its protective function via facilitating LPS recruitment and clearance although LPS down-regulates its mRNA expression [28,29]. Herein, our results showed AS had no influence on mRNA expressions of *SR C–H* of macrophages (data not shown), but AS could up-regulate *SR-A* and *MARCO* mRNA expression, and up-regulate *SR-BI* mRNA decreased by LPS, suggesting AS enhancement of LPS internalization and inhibition of pro-inflammatory cytokines was related to increasing the mRNA expression of scavenger receptors (*SR-A*, *MARCO* and *SR-BI*).

## 3. Experimental Section

### 3.1. Materials

Injectable artesunate (AS) was purchased from Guilin Nanyao LTD (Guangxi, China). Ampicillin sodium-sulbactam sodium (AMPS) was purchased from Seashore Pharmaceutical Company (Shenzhen, China). LPS O111:B4, MDC, MTT and DAPI were purchased from Sigma Chemicals (St. Louis, MO, USA). FITC-LPS was made in our lab [21]. Mouse TNF-α and IL-6 ELISA kits were purchased from R & D Systems Inc. (Minneapolis, MN, USA). ALT and AST kits were purchased from Jiancheng Institute of Biotechnology (Nanjing, China). LDH cytotoxicity assay kit was purchased from Beyotime Institute of Biotechnology (Haimen, China). PrimeScript RT reagent Kit was obtained from TAKARA (Dalian, China). qPCR Master Mix was purchased from ToYoBo Ltd (Osaka, Japan). PCR primers for *SR-A*, *SR-BI*, *MARCO* and *β-actin* were synthesized from Sangon Biotech Co., Ltd. (Shanghai, China).

### 3.2. Experimental Sepsis Induced by CLP and Survival Rate

Specific-pathogen-free (SPF) Kunming mice (4–6 weeks old, weighing 18–20 g, male and female in equal number) were obtained from the Experimental Animal Center of the Third Military Medical University (Chongqing, China). All animal experiments were performed in accordance with the National Guidelines for Animal Care and used to establish the CLP sepsis model and obtain peritoneal macrophages and liver Kupffer cells.

In the CLP model, mice were anaesthetized by ether. Approximately two third of the cecum was ligated through a 1.5-cm abdominal midline surgical incision. The ligated part of the cecum was punctured through and through with a 21-gauge needle. After repositioning of the bowel, the abdomen was closed using a surgical suture. Sham animals underwent the same procedure without ligation or puncture of the cecum. Mice were randomly divided into the following six groups (25 mice/group): group 1, CLP model group; group 2, immediately intramuscular injection with AS (30 mg/kg) after CLP; group 3, immediately intramuscular injection with AMPS (100 mg/kg) after CLP; group 4–6, CLP model and immediately intramuscular injection with AS (7.5, 15 and 30 mg/kg) and AMPS (100 mg/kg) after CLP. According to the clinical dosage regimen against malaria, AS and AMPS was repeatedly administered at 0, 4, 24, and 48 h after CLP operation. The general condition and mortality of the mice were observed for seven days.

### 3.3. Experimental Sepsis Induced by CLP and Serum Collection

Mice were randomly divided into four groups (8 mice/group): group 1, Sham group: underwent the same procedure without ligation or puncture of the cecum; group 2, CLP model group; group 3, immediate intramuscular injection with AS (15 mg/kg) after CLP operation; group 4, immediate intramuscular injection with AS (30 mg/kg) after CLP operation.

At 4 and 24 h after surgical operation respectively, 4 mice from each group were sacrificed and sera were separated from the blood samples of sacrificed mice by centrifugation at 3500× *g* for 20 min and stored at −80 °C for subsequent assays. The TNF-α and IL-6 levels were tested using respective mouse ELISA kits. The serum LPS level was assayed using the LAL test. Serum AST and ALT levels were measured using spectrophotometric diagnostic kits (Beyotime Institute of Biotechnology, Nanjing, China) according to the manufacturer’s recommendations.

### 3.4. Isolation of Peritoneal Macrophages and Liver Kupffer Cells from Mice and Cell Culture

The murine peritoneal macrophages were isolated and purified as described previously [5,30]. In order to obtain liver Kupffer cells, the liver was removed after portal vein perfusion with Hank’s balance salt solution (HBSS) for digestion in a solution of 0.1% collagenase IV, 0.4% pronase E and 0.012% Dnase I. Kupffer cells were isolated by degradient centrifugation in Percoll (Sigma Chemicals, St. Louis, MO, USA). The cells were cultured at 37 °C in a 5% CO_2_ humidified incubator and maintained in RPMI 1640 supplemented with 10% endotoxin free fetal calf serum (FCS) (HyClone; Logan, UT, USA), 100 U/mL penicillin G, and 100 μg/mL streptomycin. The cells were diluted with 0.4% trypan blue in phosphate buffered saline (PBS; 0.1 mM, pH 7.4) and live cells were counted by a hemocytometer.

### 3.5. Immunofluorescence Imaging of LPS Internalization

Cells (1.0 × 10^6^/mL) on glass coverslips were incubated in 0.5 mL serum free RPMI 1640 for 4 h. MDC (25 μM), a specific inhibitor for clathrin-dependent pathway, was added and the cells were cultured for another 1 h. Subsequently, cells were pretreated with or without AS (10 μg/mL) for 2 h, and then were further treated with FITC-LPS (500 ng/mL) for 30 min, and washed three times with warm PBS and fixed in 4% paraformaldehyde for 20 min at room temperature. Nuclei were stained with nucleic acid dye DAPI. Samples were examined by laser confocal scanning microscope (Zeiss, Jena, Germany) at appropriate wavelengths. Images were captured and processed using the LSM Image Examiner software (version 3.1.0; Carl Zeiss, Jena, Germany).

### 3.6. Cytokine Quantification

Cells (1.0 × 10^6^ cells/mL, 0.4 mL) were incubated in 48-well plates for 4 h, and supernatants were then discarded and replaced with 0.4 mL serum-free RPMI 1640 medium. The cells were co-incubated with MDC (25 μM) for 1 h. Cells were then treated with or without AS (10 μg/mL) for 2 h and then stimulated with LPS (0.1 μg/mL) for 4 h. The supernatants were collected and stored at −20 °C for analysis. The levels of TNF-α and IL-6 in the supernatants were analyzed using ELISA kits according to the manufacturers’ instructions. Briefly, ELISA plates were coated with 100 μL/well of capture antibody in coating buffer overnight at 4 °C. After blocking of the plates, 100 μL/well of test standard or sample was incubated at room temperature for 1 h. 100 μL/well of detection antibody and Avidin-HRP (R & D, Minneapolis, MN, USA) was used to detect bound samples. The wells were rinsed with wash buffer (PBS containing 0.05% Tween-20). Substrate development was stopped by stop buffer (2NH_2_SO_4_). Plate was read at 450 nm to measure the absorbance [5].

### 3.7. Quantitative PCR Analysis

Adherent cells (2.0 × 10^6^/mL, 2 mL) cultured in 6-well plates were incubated with or without AS (10 μg/mL) for 2 h. Subsequently, cells were stimulated with LPS (100 ng/mL) for 4 h. Total RNA was extracted from the harvested cells using a Trizol reagent (Invitrogen, Carlsbad, CA, USA) and reverse transcribed into cDNA with a PrimeScript RT reagent Kit (TOYOBO, Japan). The primers for *SR-A*, *MARCO*, *SR-BI* and *β-actin* (Table 1) were added to the PCR tubes. Reactions were set up for qPCR per manufacturer’s instructions containing 2 μL of cDNA, 10 μL of SYBR green mix (TOYOBO, Osaka, Japan), 1 μL of specific forward primer (10 μM stock), 1 μL of specific reverse primer (10 μM stock), and 6 μL of distilled water. Amplification reactions were performed in a 7500 Real-Time PCR System (Applied Biosystems, Foster City, CA, USA) for 40 cycles of denaturation at 94 °C for 30 s, annealing at 55 °C for 30 s and extension at 72 °C for 40 s. *C*_T_ values were determined using the 7500 System SDS Software (v.1.2.3; Applied Biosystems, Foster City, CA, USA). Expression ratios were calculated according to the 2^−△△^*^C^*^T^ method described [31]. Each sample was analyzed in quadruplicate.

### 3.8. Cytotoxicity Assays

Cytotoxicities of agents were determined using MTT or LDH assay. Cells (5.0 × 10^4^ cells/well) were plated in 96-well plates in RPMI 1640 for 4 h, and then washed twice and incubated with indicated concentrations of MDC and AS for 6 h. MTT assay: Subsequently, 20 μL of the MTT solution (5 mg/mL) was added to the medium in a total volume of 200 μL. After the cells were incubated for 4 h, the supernatant was removed, and 150 μL of DMSO was added to each well to dissolve the produced formazan crystals. The extinction was measured at 490 nm in a Model 550 microplate reader (Bio-Rad, Hercules, CA, USA). LDH assay [32]. After the cells were centrifuged at 400× *g* for 5 min, the clear supernatant (120 μL/well) was transferred into corresponding wells of an optically clear 96-well plate. Sixty μL of the LDH reaction mixture was added to each well and incubated for up to 30 min at room temperature (the plate was protected from light). The extinction was measured at 490 nm in a Model 550 microplate reader (Bio-Rad, Hercules, CA, USA). The percentage cytotoxicity was calculated according to the protocol of the manufacturer’s instructions: Cytotoxicity (%) = (Test Sample – Low Control)/(High Control – Low Control) × 100.

### 3.9. Statistical Analysis

All experiments were performed at least three times and representative data were presented. Cytokine concentrations were expressed as mean ± S.D. One-way ANOVA test was used for multiple comparisons. A *p* value less than 0.05 was considered statistically significant.

## 4. Conclusions

In conclusion, our results demonstrate that AS protects CLP sepsis mice by inhibiting pro-inflammatory cytokine release and serum LPS levels, which is tightly related to enhancement of LPS internalization of macrophage via increasing the mRNA expression of scavenger receptors (*SR-A*, *MARCO*, *SR-BI*).

## Supplementary Information



## Figures and Tables

**Table 1.** Primers used for PCR analysis of gene expression.

Gene	Primers	Product size
*SR-A*	PF: 5′-ATTGGGAAATGAAGAACTGC-3′ PR:5′-GGACTGACGAAATCAAGGAA-3′	267 bp
*SR-BI*	PF: 5′-CCACCCAACGAAGGCTTCTGC-3′ PR: 5′-CTGAATGGCCTCCTTATCC-3′	190 bp
*MARCO*	PF: 5′-AGAAAGGGAGACACTGGAAGC-3′ PR: 5′-CCTCTGGAGTAACCGAGCAT-3′	274 bp
*β-actin*	PF: 5′-GAGACCTTCAACACCCCAGC-3′ PR: 5′-ATGTCACGCACGATTTCCC-3′	263 bp
