# Supplementary material for: Artesunate Reduces Serum Lipopolysaccharide in Cecal Ligation/Puncture Mice via Enhanced LPS Internalization by Macrophages through Increased mRNA Expression of Scavenger Receptors
Source: Int J Mol Sci. 2014 Jan 16;15(1):1143–61. doi: 10.3390/ijms15011143 (PMC3907860; doi:10.3390/ijms15011143)
Supplement: Supplementary file 1 [file ijms-15-01143-s001.pdf]

Supplementary Information

Figure S1. Effect of AS on FITC-LPS internalization activity of liver Kupffer cells from CLP mice. Group 1, sham group; group 2, CLP model group; goup 3, immediate intramuscular injection with AS (30 mg/kg) after CLP. At 4 h after CLP operation, mice were scarified. Mouse liver Kupffer cells were isolated and cultured (1.0 × 10^6/mL, 500 μL) grown on glass coverslips. Cells were treated with FITC-LPS (500 ng/mL) for 30 min (FITC-LPS). After washing and fixation, the nuclei were stained with 4',6-diamidino-2-phenylindole (DAPI, blue). The distribution of FITC-LPS as green color in cells was observed by laser confocal microscope. Images were captured and processed using the LSM Image Examiner software (version 3.1.0; Carl Zeiss, Jena, Germany).

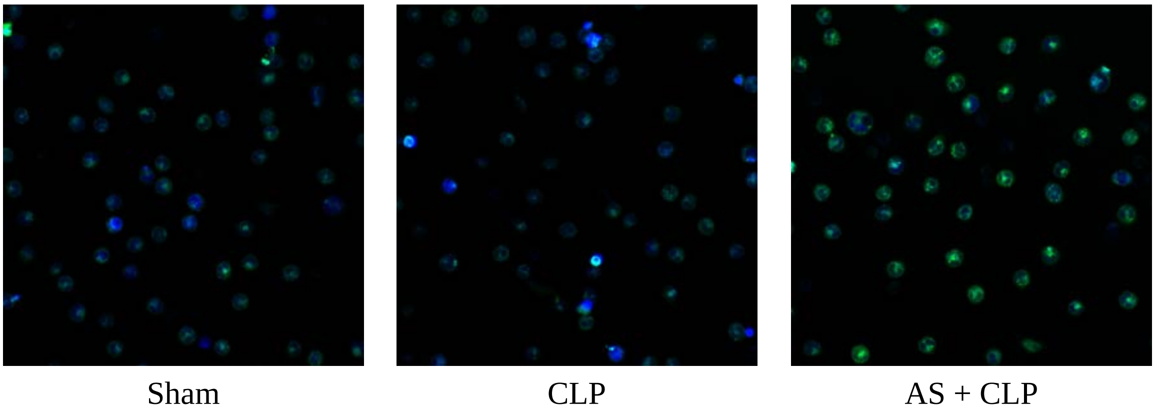

© 2014 by the authors; licensee MDPI, Basel, Switzerland. This article is an open access article distributed under the terms and conditions of the Creative Commons Attribution license (http://creativecommons.org/licenses/by/3.0/).
